# Extracellular high mobility group box 1 as a UV‐induced alarmin driving cutaneous inflammation and photoaging via TLR4 signaling

**DOI:** 10.1002/ccs3.70109

**Published:** 2026-08-27

**Authors:** Qian Liufu, Zhaoping Qin, Yan Yan, Yiru Xu, Tianyuan He, Yingchun Liu, Yaping Xiang, Chunfang Guo, Taihao Quan

**Affiliations:** ^1^ Department of Dermatology University of Michigan Medical School Ann Arbor Michigan USA; ^2^ The First Affiliated Hospital of Guangzhou Medical University Guangzhou China

**Keywords:** cytokine, HMGB1, human skin, UV

## Abstract

High mobility group box 1 (HMGB1) is a nuclear protein that functions as a damage‐associated molecular pattern molecule upon extracellular release. It has an established role in sterile inflammation, yet its involvement in UV‐induced human skin inflammation remains poorly characterized. This study investigated whether UV irradiation and chronic photoaging trigger HMGB1 release in human skin and whether this release drives proinflammatory cytokine expression and photoaging. Both acutely UV‐irradiated and chronically photoaged human skin exhibited HMGB1 release, which temporally correlated with the upregulation of photoaging‐related critical proinflammatory cytokines, IL‐1β, IL‐6, IL‐8, and TNF‐α. In cultured cells, UV irradiation reduced intracellular HMGB1 protein without altering mRNA levels while simultaneously increasing extracellular HMGB1 in supernatants, confirming active secretion rather than passive leakage. HMGB1 knockdown and blockade of TLR4, its principal receptor, significantly attenuated UV‐induced proinflammatory cytokine expression in both cell types. In mouse skin, disruption of the HMGB1–TLR4 axis reduced neutrophil infiltration, suppressed cytokine expression, and mitigated dermal damage (photoaging). Collectively, these findings demonstrate that UV irradiation promotes active HMGB1 release from skin cells, amplifying cutaneous inflammation and exacerbating dermal damage via TLR4 signaling. Therapeutic targeting of the HMGB1–TLR4 axis therefore represents a promising strategy for mitigating UV‐induced skin inflammation and its downstream pathologies, including photoaging.

## Graphical abstract

UV irradiation triggers the active release of HMGB1 from skin cell nuclei into the extracellular space. Extracellular HMGB1 binds TLR4 receptors on neighboring cells, initiating a signaling cascade that upregulates proinflammatory cytokines (IL‐1β, IL‐6, IL‐8, and TNF‐α). This chronic inflammatory state drives neutrophil infiltration and collagen fiber fragmentation in the dermis, collectively promoting photoaging and skin inflammation.

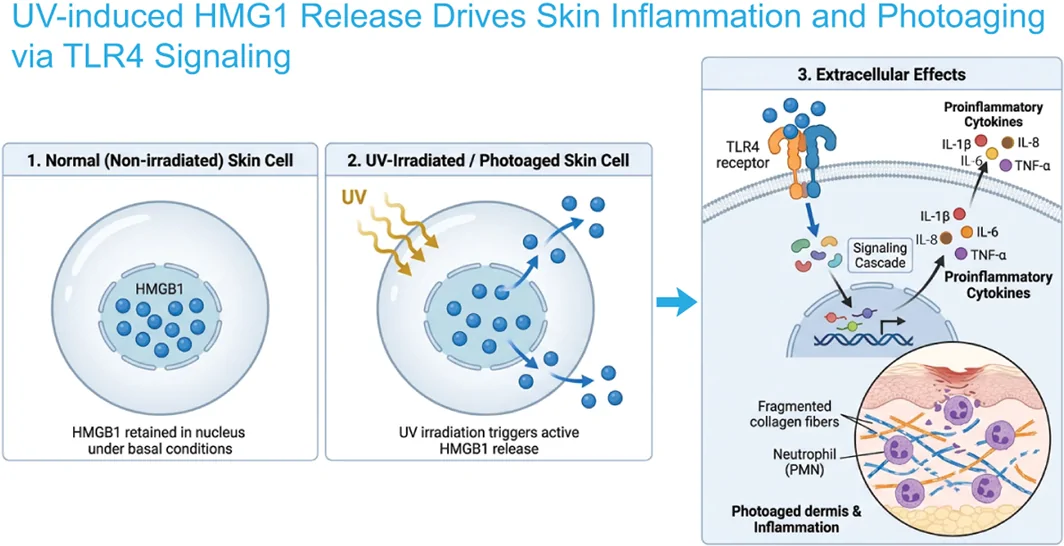

## 1 INTRODUCTION

Ultraviolet (UV) radiation is a primary environmental factor that damages human skin, contributing to photoaging,^1^ immunosuppression,^2^ and skin cancer development.^3^ Acute UV exposure triggers a cascade of inflammatory responses characterized by the production of proinflammatory cytokines, which play critical roles in both protective and pathological outcomes.^4^ Although the direct DNA‐damaging effects of UV radiation have been extensively studied, the molecular mechanisms underlying UV‐induced inflammation remain incompletely understood.

High mobility group box 1 (HMGB1) is a ubiquitous nuclear protein that functions both as a chromatin‐binding factor and as a damage‐associated molecular pattern (DAMP) molecule.^5^, ^6^ Under physiological conditions, HMGB1 resides predominantly in the nucleus, where it functions as a nonhistone chromatin‐associated protein that plays a central role in maintaining genomic integrity and regulating gene expression.^7^ Under conditions of cellular stress or damage, HMGB1 undergoes a functional transition, shifting from its role as a nuclear protein to that of an extracellular signaling mediator through either active secretion or passive release. Once in the extracellular space, HMGB1 exerts cytokine‐like effects by engaging pattern recognition receptors, most notably toll‐like receptors (TLRs) and the receptor for advanced glycation end products (RAGE), thereby triggering downstream inflammatory signaling cascades.^8^, ^9^ The contribution of HMGB1 as a central mediator of sterile inflammation has been well documented across a spectrum of pathological conditions, encompassing ischemia‐reperfusion injury, autoimmune diseases, and tissue damage.^6^, ^10^ However, the involvement of HMGB1 in UV‐induced human skin inflammation has not been thoroughly investigated. Given that UV irradiation causes significant cellular stress and damage in skin, and that HMGB1 release is a hallmark of cellular injury, we hypothesized that UV exposure induces HMGB1 release from skin cells, which subsequently contributes to the proinflammatory cytokine response characteristic of UV‐damaged skin.

In this study, we demonstrate that UV irradiation triggers HMGB1 release from both epidermal keratinocytes and dermal fibroblasts, the two major cell types in skin, and that this release temporally correlates with the induction of key proinflammatory cytokines. Furthermore, we provide evidence that HMGB1–TLR4 signaling contributes to UV‐induced cytokine expression, suggesting that targeting this pathway may represent a therapeutic strategy for mitigating UV‐induced skin inflammation and potentially preventing photoaging and other UV‐related skin damage.

## 2 MATERIALS and METHODS

### 2.1 Human skin tissue analysis, UV exposure, quantitative real‐time RT‐PCR, and immunohistology

All procedures involving human individuals were approved by the University of Michigan Institutional Review Board, and all individuals provided written informed consent in adherence to the Declaration of Helsinki Principles (see ethics approval for details). For UV exposure experiments, sun‐protected buttock skin from human subjects (average age 39 ± 8.2 years, three male and three female, *N* = 6) received 2 minimal erythema dose (MED, corresponded to approximately 0.7–1.1 J/cm^2^ UVA or 50–80 mJ/cm^2^ UVB) of solar‐simulated UV radiation.^11^ Solar‐simulated UV irradiation was delivered using a SPEC 450 W xenon arc solar simulator, which generated a spectrum composed of 77.2% UVA1 (340–400 nm), 15.9% UVA2 (320–340 nm), and 6.9% UVB (290–320 nm), yielding a UVA/UVB ratio of 13.5:1. Before each exposure, irradiance was calibrated using a UV radiometer (ILT1400 A, InternationalLight) to ensure accurate and reproducible UV dosing. Individual MED values, defined as the UV dose producing minimal skin reddening, were determined for each participant 24 h post‐exposure. Skin biopsies were obtained at specified time intervals following UV treatment. Nonirradiated control skin samples were collected at the conclusion of the UV exposure period for comparative analysis. Photoaged skin biopsies were collected from the extensor forearm, whereas sun‐protected skin samples were taken from the underarm of the same individuals. Study participants with photoaged skin ranged from 54 to 65 years old. Severe photodamage was assessed using previously described clinical criteria.^12^ Total RNA extraction from skin biopsies was performed using the RNeasy Micro kit (Qiagen, Chatsworth, CA, USA). Amplified cDNA quality and concentration were evaluated with an Agilent 2100 bioanalyzer (Agilent Technologies). Cytokine transcript quantification was accomplished using the TaqMan PreAmp Master Mix kit (Applied Biosystems). PCR primers for all target genes were sourced from Real Time Primers, LLC (Elkins Park). Target gene mRNA expression levels were normalized against the housekeeping gene 36B4, which functioned as an internal reference control. Immunohistological analysis followed established protocols.^13^ Briefly, OCT‐embedded skin specimens were cryosectioned at 7 μm thickness, fixed in 2% paraformaldehyde, and permeabilized using 0.5% Triton X‐100 in phosphate‐buffered saline (PBS). Following blocking with 5% serum in PBS, sections were incubated with primary antibodies, HMGB1 (Abcam, Cell Signaling Technology), HMGB2 (Abcam), neutrophil LY‐6G (Cell Signaling Technology) for 1 h at room temperature, and then with appropriate secondary antibodies for an additional hour at room temperature. Between procedural steps, slides underwent 10‐min washes in Tris‐buffered saline containing 0.1% Triton X‐100 (TBST). Staining specificity was verified by substituting primary antibodies with corresponding isotype‐matched control immunoglobulins. The intensity of the immunostaining was quantified using Image J (FIJI‐Win64, NIH).

### 2.2 Cell culture and UV irradiation

Primary adult human keratinocytes and dermal fibroblasts were obtained from skin biopsies of healthy buttock tissue from donors aged 25–52 years, following established protocols.^14^ Cells were maintained at 37°C in a humidified atmosphere containing 5% CO_2_. Keratinocytes were grown in keratinocyte medium, whereas fibroblasts were cultured in Dulbecco's modified Eagle's medium containing 10% fetal bovine serum (Invitrogen, Carlsbad, CA), penicillin (100 U/mL), and streptomycin (100 μg/ml). Cells were seeded at 70%–80% confluence and used the next day. UV irradiation was carried out according to previously described methods.^15^ In brief, sub‐confluent cell cultures were irradiated with UVB at 50 mJ/cm^2^ using an Ultralite Panelite lamp equipped with six FS24T12 UVB‐HO bulbs. A Kodacel TA410/407 filter (Eastman Kodak) was employed to eliminate UVC wavelengths (below 290 nm). UV irradiation intensity was measured using an IL400 A phototherapy radiometer equipped with a SED240/UVB/W photodetector (InternationalLight, Newbury, MA). For HMGB1 knockdown experiments, cells were transiently transfected with HMGB1 siRNA (Santa Cruz) using electroporation (Amaxa) and incubated for 48 hours. For TAK‐242 (Sigma) treatment, cells were pretreated with TAK‐242 (100 μM) for 1 h prior to UV irradiation.

### 2.3 Western blot analysis and ELISA

Cellular proteins were extracted using whole cell lysis buffer. Protein samples (∼100 μg) were resolved by 12% SDS‐PAGE and electrotransferred to PVDF membranes. Membranes were blocked for 1 hour at room temperature in PBST (PBS containing 0.1% Tween 20) supplemented with 5% nonfat milk. The membranes were then probed with primary antibodies (HMGB1, Abcam; p65 and p‐ERK, Cell Signaling Technology, and *β*‐actin, Sigma, St. Louis, MO, USA) for 1 h at room temperature. Following three washes in PBST, membranes were incubated with corresponding horseradish peroxidase‐conjugated secondary antibodies for 1 hour at room temperature. After an additional three PBST washes, protein bands were visualized using the Vistra ECF Western blotting system (GE HealthCare) according to the manufacturer's protocol. Membrane images were captured with a STORM PhosphorImager (Molecular Dynamics), and band intensities were quantified using ImageQuant software (GE HealthCare). Protein expression levels were normalized to *β*‐actin to ensure equal loading. The HMGB1 ELISA kit was purchased from R&D Systems and used according to the manufacturer's instructions.

### 2.4 Mouse experiment, second harmonic generation (SHG) microscopy, and collagen degradation

Eight‐week‐old female C57BL/6J mice (strain #000664) were obtained from the Jackson Laboratory. All animal experiments were reviewed and approved by the Unit for Laboratory Animal Medicine (ULAM) at University of Michigan (see ethics approval for details). Prior to UV exposure, mice received topical application of TAK‐242 (3 mg/kg) and were treated for two hours. Subsequently, the mice were irradiated with UVB light (150 mJ/cm^2^), and dorsal skin samples were collected for analysis. SHG images were acquired using a Leica SP8 Confocal Microscope 2‐Photon (laser excitation wavelength: 800 nm, recorded emission wavelength: 400 nm) at the University of Michigan Microscopy and Image Analysis Laboratory. To account for spatial heterogeneity, we analyzed multiple fields of view per sample. Raw SHG images were processed using ImageJ software (NIH, FIJI‐Win64), with background signal was subtracted using measurements from adjacent regions devoid of visible collagen, and pixel intensity values were calibrated to quantify collagen density. Collagen degradation was determined by collagen hybridizing peptide (CHP) (3Helix Inc.), which specifically binds denatured and unfolded collagen chains. OCT‐embedded skin sections were stained with CHP (conjugated to a fluorescent dye, 5‐FAM) and imaged using fluorescence microscopy.

### 2.5 Statistical analysis

Data are expressed as mean ± SEM. Statistical analyses were performed using Prism ANOVA. All *p* values are considered significant when <0.05.

## 3 RESULTS

### 3.1 UV irradiation induces HMGB1 release and that correlates with UV‐induced cytokine expression in human skin

**FIGURE 1:**
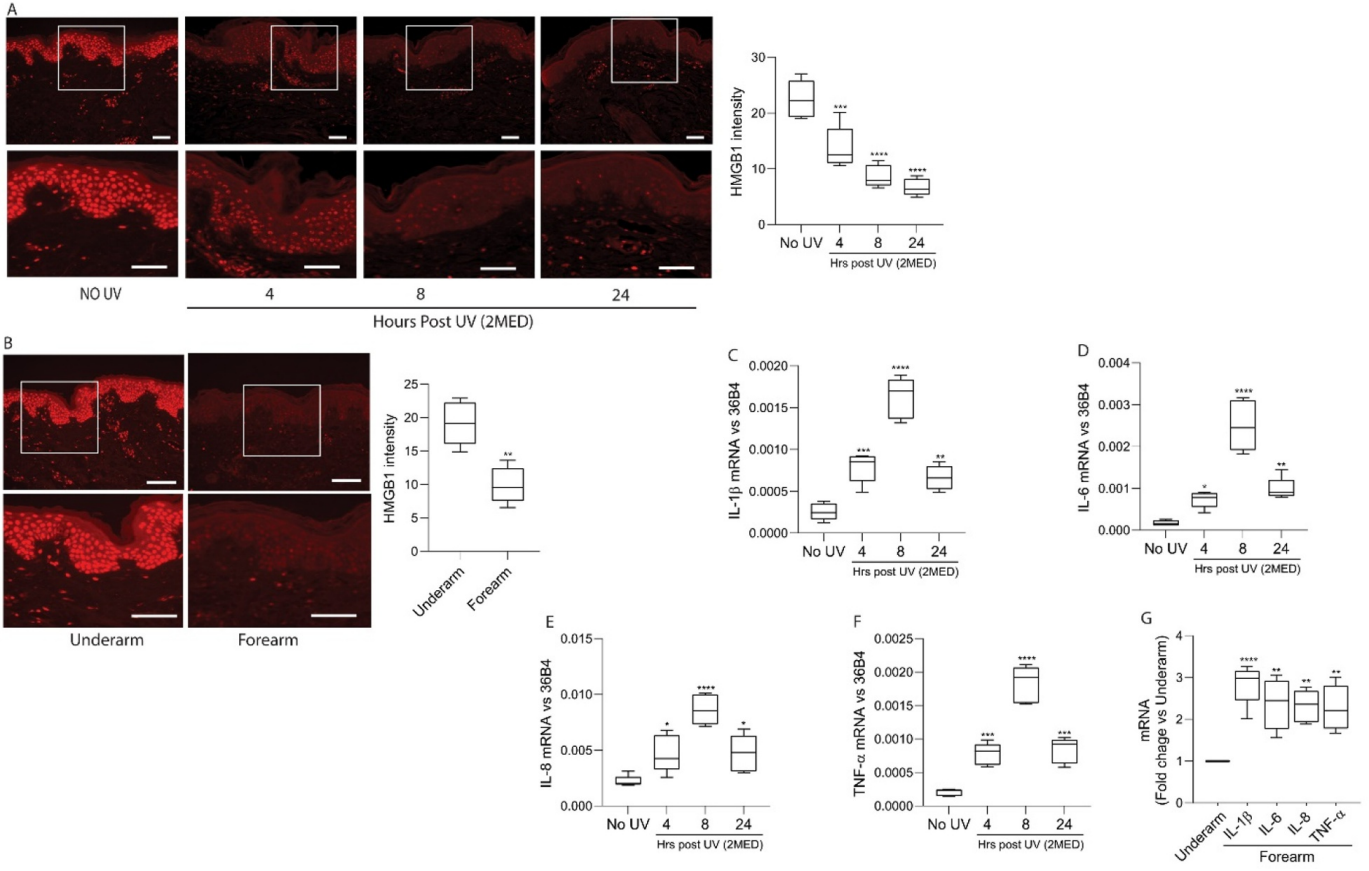
UV irradiation induces HMGB1 release and correlates with UV‐induced cytokine production in human skin. (A) Representative immunofluorescence staining of HMGB1 in sun‐protected buttock skin following acute UV irradiation (2MED). Scale bar = 100 μm. (B) HMGB1 immunofluorescence staining in chronically photoaged forearm skin compared to sun‐protected underarm skin. Scale bar = 100 μm. (C–F) Time course analysis of proinflammatory cytokine expression following UV irradiation; IL‐1β (C), IL‐6 (D), IL‐8 (E), and TNF‐α (F). (G) Comparison of proinflammatory cytokine levels in chronically photodamaged forearm skin versus sun‐protected underarm skin. mRNA levels were quantified by qRT‐PCR and normalized to 36B4 housekeeping gene. Data represent mean ± SEM from six individuals. *p < 0.05, **p < 0.01, ***p < 0.001, ****p < 0.0001 versus baseline. HMGB1, high mobility group box 1.

To determine the impact of acute UV irradiation on HMGB1 in human skin, we exposed sun‐protected buttock skin to UV and obtained biopsies at 4, 8, and 24 h post‐irradiation. HMGB1 immunofluorescence staining revealed that UV irradiation significantly reduced HMGB1 expression in both epidermal keratinocytes and dermal fibroblasts in a time‐dependent manner (Figure 1A). To confirm these findings, skin sections were immunostained with an alternative HMGB1 antibody, which demonstrated that UV irradiation significantly reduced HMGB1 expression in the skin (Supporting Information Figure S1). Consistent with these findings, HMGB1 expression was reduced in chronically photoaged human skin (Figure 1B). To assess whether UV‐induced HMGB1 release corresponded to proinflammatory cytokine production, we determined the expression of classic proinflammatory cytokines (IL‐1β, IL‐6, IL‐8, and TNF‐α). UV irradiation induced expression of these cytokines at 4 hours, with peak levels occurring at 8 hours post‐irradiation (Figure 1C–F). These proinflammatory cytokines were similarly upregulated in photodamaged forearm skin compared to sun‐protected underarm skin (Figure 1G). These results demonstrate that UV irradiation induces HMGB1 release that correlates temporally with proinflammatory cytokine expression in human skin.

### 3.2 UV irradiation induces HMGB1 release in human skin keratinocytes and fibroblasts

**FIGURE 2:**
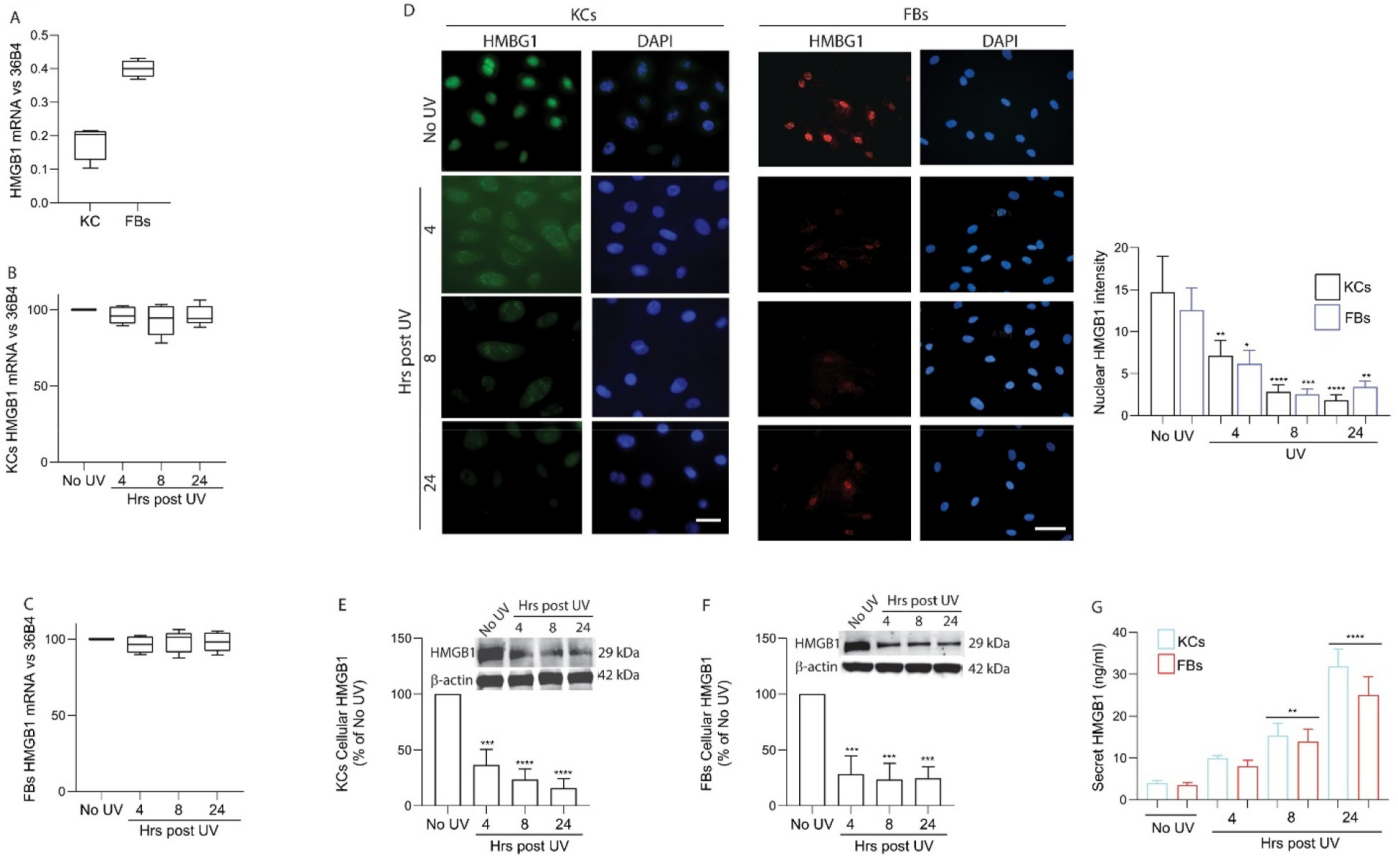
UV irradiation induces HMGB1 secretion in keratinocytes and fibroblasts. (A) HMGB1 mRNA expression in KCs and FBs. (B, C) HMGB1 mRNA expression in keratinocytes (B) and fibroblasts (C) after UV irradiation (50 mJ/cm^2^). mRNA levels were quantified by qRT‐PCR and normalized to 36B4 housekeeping gene. (D) Immunofluorescence microscopy showing HMGB1 localization (green in KCs and red in FBs) and nuclear DAPI staining (blue) after UV irradiation. Scale bar = 50 μm. (E, F) Cellular HMGB1 protein levels in keratinocytes (E) and fibroblasts (F) after UV irradiation. Representative Western blots are shown with *β*‐actin as loading control. (G) Time course of HMGB1 secretion into culture supernatants from keratinocytes and fibroblasts following UV irradiation. HMGB1 levels were measured by ELISA. Data represent mean ± SEM from at least three independent experiments. ***p < 0.001, ****p < 0.0001 versus No UV. KCs, keratinocytes; FBs, fibroblasts; HMGB1, high mobility group box 1.

As keratinocytes and fibroblasts are the two major cell types in human skin, we assessed the time‐course of HMGB1 expression in these cells following UV irradiation. Endogenous HMGB1 mRNA expression was substantially higher in fibroblasts compared to keratinocytes (Figure 2A), and UV irradiation did not alter HMGB1 transcription in either keratinocytes (Figure 2B) or fibroblasts (Figure 2C). However, immunofluorescence staining revealed that HMGB1 protein levels were significantly reduced in UV‐irradiated keratinocytes and fibroblasts as early as 4 h post‐UV and remained reduced through 24 hours post‐irradiation (Figure 2D). Western blot analysis confirmed that HMGB1 was similarly reduced in both keratinocytes (Figure 2E) and fibroblasts (Figure 2F) following UV irradiation. To determine whether the reduction in cellular HMGB1 was due to protein release, HMGB1 levels in culture supernatants were analyzed by ELISA. HMGB1 levels were increased in response to UV in both keratinocytes and fibroblasts in a time‐dependent manner (Figure 2G). Together, the loss of HMGB1 protein in cell lysates and the concomitant increase in HMGB1 in culture supernatants demonstrate that UV irradiation induces HMGB1 release from both keratinocytes and fibroblasts.

### 3.3 UV‐induced proinflammatory cytokine induction was reduced by inhibiting HMGB1–TLR interaction

**FIGURE 3:**
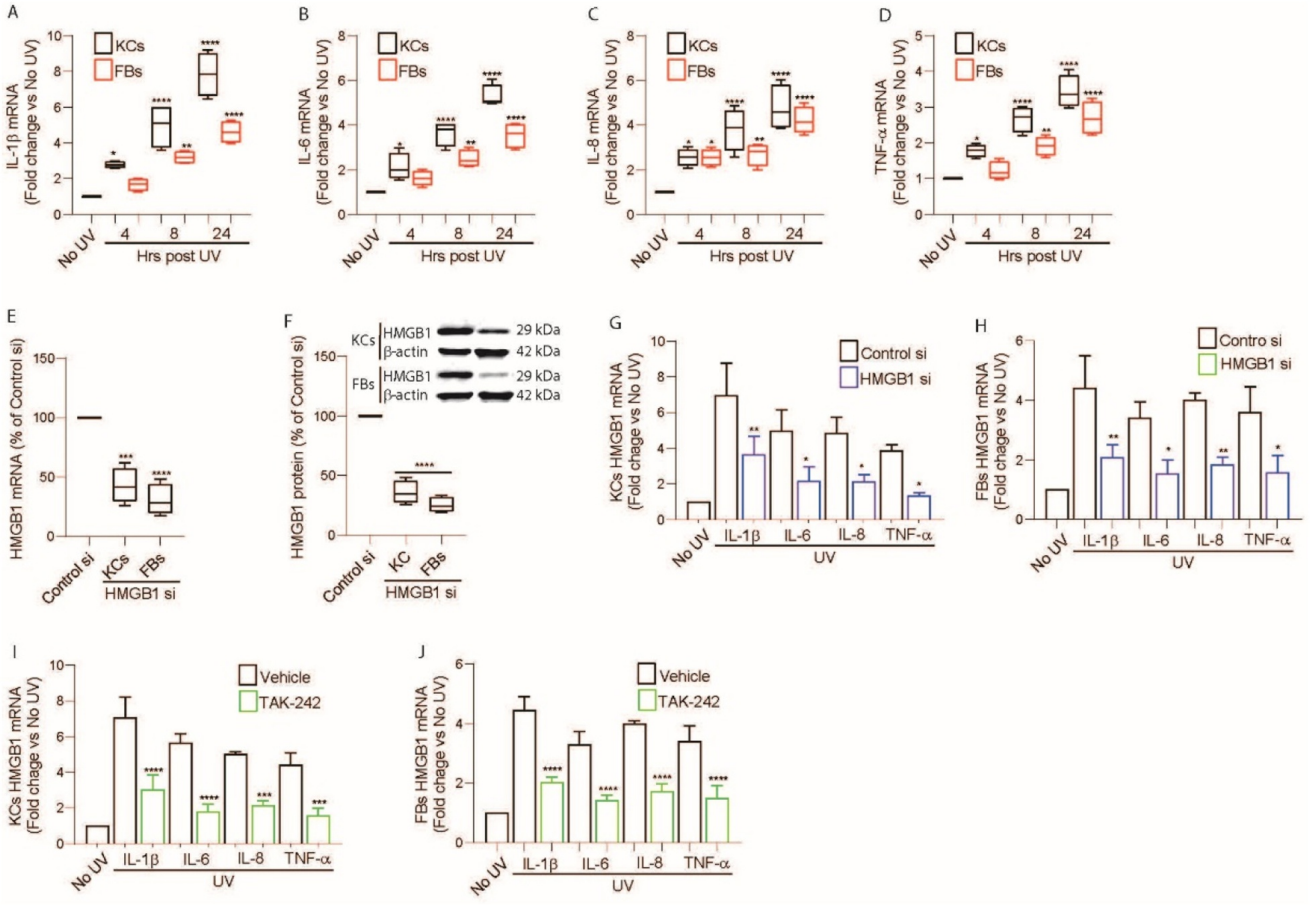
Expression of UV‐induced proinflammatory cytokine is mediated by the HMGB1–TLR4 pathway. (A–D) Time course of proinflammatory cytokine mRNA expression following UV exposure in keratinocytes (KCs) and fibroblasts (FBs). (A) IL‐1β, (B) IL‐6, (C) IL‐8, and (D) TNF‐α. (E–H) HMGB1 knockdown efficiency in KCs and FBs. (E) HMGB1 mRNA and (F) protein levels were assessed by qRT‐PCR and Western blot, respectively. Representative Western blots show HMGB1 and *β*‐actin (loading control). (G, H) HMGB1 knockdown reduces UV‐induced proinflammatory cytokine expression. (I, J) TLR4 inhibition by TAK‐242 suppresses UV‐induced cytokine expression in KCs (I) and FBs (J). mRNA levels were quantified by qRT‐PCR and normalized to 36B4 housekeeping gene. Data represent mean ± SEM from at least three independent experiments. *p < 0.05, **p < 0.01, ***p < 0.001, ****p < 0.0001. HMGB1, high mobility group box 1.

To determine whether UV‐induced HMGB1 release corresponded to proinflammatory cytokine production, we first measured levels of proinflammatory cytokines (IL‐1β, IL‐6, IL‐8, and TNF‐α) in both keratinocytes and fibroblasts. UV irradiation induced IL‐1β (Figure 3A), IL‐6 (Figure 3B), IL‐8 (Figure 3C), and TNF‐α (Figure 3D) in both cell types, with lower magnitude responses in fibroblasts compared to keratinocytes.

Next, we investigated the role of HMGB1 in UV‐induced cytokine expression. HMGB1 was knocked down in both keratinocytes and fibroblasts, and knockdown efficiency was confirmed at both the mRNA (Figure 3E) and protein (Figure 3F) levels. Notably, UV‐induced proinflammatory cytokine expression was partially reduced by HMGB1 knockdown in both keratinocytes (Figure 3G) and fibroblasts (Figure 3H).

Because HMGB1 binds to DAMP receptors such as TLRs to trigger inflammatory responses,^16^ we assessed whether blocking the HMGB1–TLR4 interaction could reduce UV‐induced proinflammatory cytokine production. To this end, we utilized a well‐characterized TLR4 inhibitor (TAK‐242) to block HMGB1 action by treating cells with TAK‐242 and examined its impact on proinflammatory cytokine expression. We confirmed that TAK‐242 efficiently blocked HMGB1‐induced proinflammatory cytokine expression in both keratinocytes (Figure 3I) and fibroblasts (Figure 3J), suggesting that secreted HMGB1 triggers TLR4 signaling, which contributes to UV‐induced cytokine production.

### 3.4 UV‐induced proinflammatory cytokine induction was reduced by inhibiting HMGB1–TLR pathway in mouse skin

**FIGURE 4:**
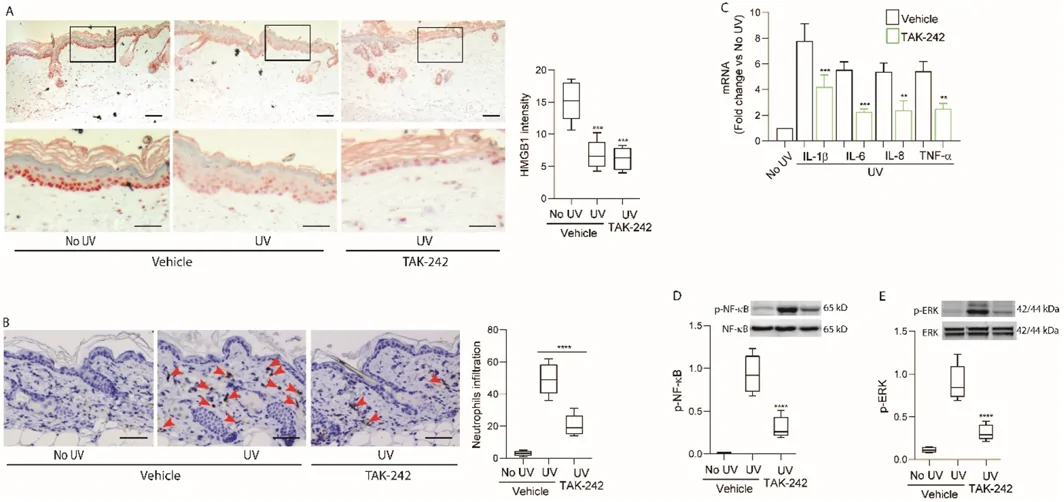
UV irradiation induces proinflammatory cytokines via the HMGB1–TLR4 axis. Mice were pretreated with vehicle (DMSO) or TAK‐242 (3 mg/kg) for two hours prior to UV irradiation (150 mJ/cm^2^) and analyzed 24 h post‐exposure. (A) Representative high mobility group box 1 immunohistochemistry. ***p < 0.001 compared to No‐UV. Scale bar = 100 μm. (B) Neutrophil infiltration assessed by Ly6G immunostaining. ****p < 0.0001 compared to vehicle‐treated UV mice. Scale bar = 100 μm. (C) mRNA expression of proinflammatory cytokines (IL‐1β, IL‐6, IL‐8, and TNF‐α). (D) NF‐κB (p65) and (E) ERK (pERK) activation. mRNA levels were quantified by qRT‐PCR and normalized to 36B4. Protein levels were determined by Western blot and normalized to *β*‐actin. Data represent mean ± SEM (*n* = 5 per group). ***p < 0.001; ****p < 0.0001 compared to vehicle‐treated UV mice.

To confirm the above in vitro findings in vivo, mice were topically treated with TK‐242 and UV‐induced cytokine levels were determined in skin. We confirmed that UV irradiation (150 mj/cm^2^) reduced the cellular expression of HMGB1 (Figure 4A), similar to observations in human skin (Figure 1). As neutrophil infiltration is the earliest immune cell response to UV irradiation, we confirmed that TAK‐242 treatment significantly attenuated neutrophil recruitment (Figure 4B). Notably, although UV irradiation significantly induced proinflammatory cytokines (IL‐1β, IL‐6, IL‐8, and TNF‐α), TLR4 inhibitor treatment resulted in suppression of these cytokine responses (Figure 4C). As HMGB1–TLR4 signaling triggers inflammatory signaling cascades through NF‐κB and MAPK pathways,^17^ we found that TAK‐242 treatment significantly attenuated the activation of NF‐κB (p65/RelA, Figure 4D) and ERK (p‐ERK, Figure 4E). Collectively, these data demonstrate that HMGB1–TLR4 signaling is a critical mediator of UV‐induced proinflammatory cytokine production in mouse skin.

### 3.5 HMGB1–TLR pathway inhibition mitigated UV‐induced dermal structural alterations in mouse skin

**FIGURE 5:**
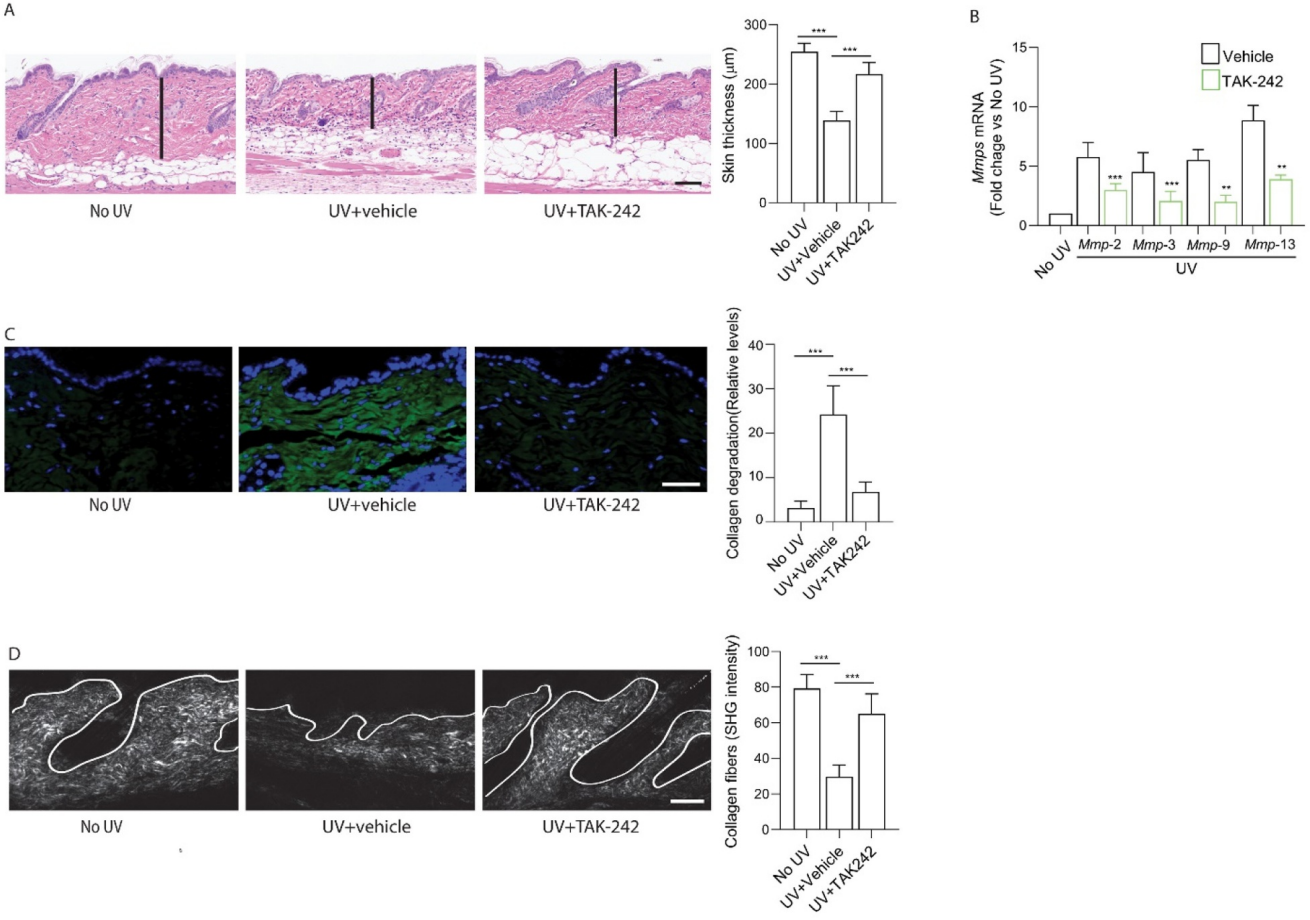
HMGB1–TLR pathway inhibition mitigated UV‐induced dermal structural alterations in mouse skin. Mice were pretreated with vehicle (DMSO) or the TLR4 inhibitor TAK‐242 (3 mg/kg) two hours prior to UV irradiation (150 mJ/cm^2^), treated twice weekly for four weeks. (A) Representative H&E‐stained sections. Bars denote epidermal/dermal thickness, quantified using Aperio ImageScope v12.1 (Leica, Deer Park, IL). Scale bar = 100 μm. (B) Dermal mRNA expression of matrix metalloproteinases (*Mmps*), quantified by qRT‐PCR and normalized to *36B4*. (C) Collagen degradation determined by collagen hybridizing peptide staining, which selectively binds denatured and unfolded collagen chains (green), quantified using ImageJ (FIJI‐Win64, NIH). Scale bar = 100 μm. (D) Collagen fiber architecture visualized by second harmonic generation imaging acquired on a Leica SP8 confocal microscope and quantified using ImageJ (FIJI‐Win64, NIH). Scale bar = 100 μm. For all panels, data represent mean ± SEM; *N* = 5 per group; **p < 0.002, ***p < 0.001.

Given that the HMGB1–TLR pathway drives UV‐induced cytokine production, we investigated whether blocking this pathway could protect against UV‐induced skin damage (photoaging). To this end, TAK‐242 was applied topically to mouse skin prior to UV irradiation (150 mJ/cm^2^, twice weekly) for four weeks. Notably, UV irradiation‐induced skin thinning, an indicator of dermal collagen loss (photoaging), was ameliorated by TAK‐242 treatment (Figure 5A). Quantitative analysis confirmed a significant preservation of skin thickness in TAK‐242‐treated mice (Figure 5A, bar graph). The TAK‐242 application also reduced UV‐induced immune cell infiltration (Figure 5A).

Based on the protective effects of TAK‐242 described above, we next examined the expression of mouse *Mmps*, which are involved in collagen degradation.^18^ Indeed, TAK‐242 inhibited UV‐induced upregulation of multiple *Mmps* (Figure 5B), and this was further confirmed by reduced collagen degradation assessed using a CHP (Figure 5C), which selectively binds to degraded collagen.^19^

SHG microscopy, which provides high‐resolution three‐dimensional (3D) images of collagen fibers, revealed that collagen bundles in nonirradiated control mice appeared thick, densely packed, tightly interwoven, and evenly distributed throughout the dermis (Figure 5D, left). In contrast, collagen bundles in UV‐irradiated skin were significantly reduced (Figure 5D, middle), an effect that was largely reversed by TAK‐242 treatment (Figure 5D, right). Quantitative analysis demonstrated a significant preservation of collagen fiber intensity in TAK‐242‐treated mice. Collectively, these data indicate that the inhibition of the HMGB1–TLR pathway attenuates UV‐induced skin damage in mice.

## 4 DISCUSSION

The present study demonstrates that UV irradiation induces HMGB1 release from human skin cells and that this released HMGB1 interacts with TLR4 to promote proinflammatory cytokine expression. Our findings establish a novel mechanism by which UV radiation triggers sterile inflammation in skin, identifying the HMGB1–TLR4 axis as a potential therapeutic target for preventing UV‐induced skin damage.

Several key findings support this conclusion. First, UV irradiation caused a rapid reduction in HMGB1 protein levels in both epidermal keratinocytes and dermal fibroblasts in human skin, with decreased levels evident as early as 4 hours post‐exposure. This reduction was not due to decreased transcription, as HMGB1 mRNA levels remained unchanged following UV exposure, indicating a post‐transcriptional mechanism of protein loss. The simultaneous detection of increased HMGB1 in culture supernatants confirmed that the decrease in cellular HMGB1 resulted from active protein release rather than degradation. This temporal pattern of HMGB1 release closely correlated with the induction of proinflammatory cytokines, which peaked at 8 h post‐irradiation, suggesting a causal relationship between HMGB1 release and cytokine production.

The functional significance of HMGB1 release was demonstrated through both loss‐of‐function and pharmacological inhibition experiments. HMGB1 knockdown partially attenuated UV‐induced cytokine expression in both keratinocytes and fibroblasts, indicating that HMGB1 contributes to, but does not exclusively mediate, the inflammatory response to UV radiation. The incomplete suppression of cytokine production by HMGB1 knockdown suggests that multiple parallel pathways contribute to UV‐induced inflammation, which is consistent with the complex nature of cellular responses to UV stress. These parallel pathways likely include direct activation of stress‐activated protein kinases, NF‐κB signaling through reactive oxygen species, and other DAMP molecules released upon UV‐induced cellular damage.

Our demonstration that TLR4 inhibition significantly reduced UV‐induced cytokine production both in vitro and in vivo provides strong evidence that extracellular HMGB1 signals through TLR4 to amplify the inflammatory response. Although HMGB1 can bind to multiple receptors including RAGE and TLR2,^8^ our data indicate that TLR4 is a critical receptor mediating HMGB1‐induced inflammation in UV‐damaged skin. The effectiveness of TLR4 inhibition in suppressing cytokine production suggests that blocking HMGB1–TLR4 signaling could provide therapeutic benefit without completely eliminating the inflammatory response, which may be important for maintaining beneficial aspects of UV‐induced inflammation such as DNA repair responses and immune surveillance.

The observation that HMGB1 expression was also reduced in chronically photoaged skin extends the relevance of our findings beyond acute UV exposure. This suggests that repeated UV‐induced HMGB1 release may contribute to the chronic low‐grade inflammation characteristic of photoaged skin. Cumulative HMGB1 release over time could perpetuate inflammatory signaling cascades that drive collagen degradation, elastin accumulation, and other hallmarks of photoaging. Thus, the HMGB1–TLR4 axis may represent not only an acute response to UV damage but also a mechanism underlying chronic photodamage.

An intriguing aspect of our findings is the differential baseline expression of HMGB1 between keratinocytes and fibroblasts, with fibroblasts expressing substantially higher levels of HMGB1 mRNA. Despite this difference, both cell types released HMGB1 in response to UV irradiation and exhibited cytokine induction upon HMGB1–TLR4 signaling. The higher basal HMGB1 expression in fibroblasts may reflect the greater nuclear activity and chromatin remodeling requirements in these metabolically active cells. The magnitude of cytokine response was greater in keratinocytes compared to fibroblasts, which may reflect differences in pattern recognition receptor expression, downstream signaling components, or the relative sensitivity of these cell types to HMGB1 stimulation. We also found that fibroblasts exhibited substantially higher HMGB2 transcription compared to keratinocytes (Supporting Information Figure S2A), similar to the pattern observed for HMGB1. Furthermore, UV irradiation reduced HMGB2 expression in human skin (Supporting Information Figure S2B) in a pattern similar to HMGB1, though to a lesser extent. Although HMGB2 has been reported to share structural and functional similarities with HMGB1 and may participate in inflammatory responses under certain conditions,^20^ its extracellular functions and potential interaction with HMGB1 in UV‐induced skin injury remain largely unknown. Our finding suggests that the concurrent release of both HMGB1 and HMGB2 following UV irradiation may act synergistically to amplify DAMP signaling and thus represent an interesting direction for future investigation.

Several questions remain to be addressed in future studies. The precise mechanism by which UV irradiation triggers HMGB1 release is not fully elucidated. UV irradiation may induce active HMGB1 secretion through several nonmutually exclusive mechanisms. These include post‐translational modifications, such as acetylation, which facilitate HMGB1 nuclear export, inflammasome‐associated pathways including pyroptosis, and extracellular vesicle/exosome‐mediated release. Any or all these mechanisms may contribute to the early extracellular accumulation of HMGB1 observed following UV exposure. HMGB1 can also be released through both active secretion via nonclassical pathways and passive release from damaged or dying cells. Our observation that HMGB1 appeared in culture supernatants at 8–24 h post‐UV, a timeframe that precedes extensive cell death in our experimental conditions, suggests active secretion as the primary mechanism. However, passive release from cells undergoing UV‐induced apoptosis or necroptosis may also contribute, particularly at later timepoints or higher UV doses. Further investigation into the specific cellular processes regulating HMGB1 translocation and secretion following UV exposure would provide valuable mechanistic insights.

The biological activity of HMGB1 is critically regulated by its redox state, with the fully reduced form primarily driving chemotaxis, whereas the disulfide form preferentially promotes proinflammatory cytokine production.^21^ UV irradiation generates reactive oxygen species that may alter the redox state of extracellular HMGB1, thereby influencing its receptor interactions and downstream signaling. Although we did not determine the redox status of HMGB1 released following UV exposure in the present study, the observed induction of inflammatory cytokines suggests the involvement of the disulfide HMGB1 isoform. Future studies employing redox‐sensitive biochemical analyses or mass spectrometry‐based approaches will be required to distinguish reduced and disulfide HMGB1 isoforms after UV exposure and to define their respective contributions to UV‐induced skin inflammation. As such, characterizing the redox state of UV‐induced HMGB1 release and its functional consequences would enhance our understanding of how HMGB1 mediates specific inflammatory responses in UV‐damaged skin.

The therapeutic implications of our findings are significant. Current approaches to preventing UV‐induced skin damage focus primarily on physical sun protection and antioxidant supplementation.^22^ Our identification of the HMGB1–TLR4 axis as a mediator of UV‐induced inflammation suggests that pharmacological intervention targeting this pathway could provide additional protection. Building on this mechanistic framework, Zhong et al. provided compelling evidence that the HMGB1–TLR4 signaling axis plays a pivotal role in malignant mesothelioma progression by shaping the tumor immune microenvironment; pharmacological disruption of this axis using TAK‐242 significantly attenuated tumor burden.^23^ These findings carry broader therapeutic implications, selective TLR4 inhibitors, and HMGB1‐neutralizing antibodies, and small molecules designed to interfere with HMGB1‐receptor binding represent promising candidate strategies that warrant investigation as topical interventions for mitigating UV‐driven cutaneous inflammation. In this study, we demonstrate that the topical application of TAK‐242, a selective inhibitor of TLR4 signaling, significantly attenuated UV‐induced proinflammatory cytokine production (Figure 4), suppressed the upregulation of multiple MMPs (Figure 5B), and reduced collagen degradation (Figure 5C,D) in mouse skin. These findings suggest that pharmacological inhibition of TLR4 signaling may represent a promising topical therapeutic strategy for alleviating UV‐induced inflammatory responses and extracellular matrix remodeling associated with skin injury and photoaging. Although TAK‐242 is widely characterized as a selective TLR4 inhibitor, previous studies have suggested that at high concentrations, it may influence the activity of other TLR family members or related innate immune pathways.^24^, ^25^ We examined whether TAK‐242 treatment itself affected the expression of TLRs and the receptor for advanced glycation end products (RAGE), another pattern‐recognition receptor involved in innate immune and inflammatory signaling. Topical TAK‐242 administration did not significantly alter the basal or UV‐induced mRNA expression of TLR2, TLR3, TLR4, or RAGE in the cells and mouse skin (Supporting Information: Figure S3), indicating that TAK‐242 does not exert detectable off‐target effects on these pattern recognition receptors under the experimental conditions.

## 5 CONCLUSION

Our study establishes that UV irradiation induces HMGB1 release from human skin cells and that this HMGB1 contributes to proinflammatory cytokine production through TLR4 signaling, and blocking the HMGB1‐TLR4 pathway attenuated skin damage following UV exposure.

These findings identify a novel mechanism underlying UV‐induced skin inflammation and suggest that targeting the HMGB1–TLR4 axis represents a promising therapeutic strategy for preventing photoaging and potentially reducing the risk of UV‐associated skin pathologies.

## AUTHOR CONTRIBUTIONS

Taihao Quan Designed and supervised the studies, analyzed data, provided funding sources and wrote the manuscript. Liufu Qian conducted human skin experiments, acquired data and analyzed data. Zhaoping Qin conducted experiments, acquired data and analyzed data. Tianyuan He, Yiru Xu, Yan Yan, Yingchun Liu, Yaping Xiang, and Chunfang Guo conducted experiments and acquired data.

## CONFLICT OF INTEREST STATEMENT

The authors declare no conflicts of interest.

## ETHICS STATEMENT

All procedures involving human individuals were approved by the University of Michigan Institutional Review Board (#HUM00139214, April 8, 2022), and all individuals provided written informed consent in adherence to the Declaration of Helsinki Principles. All animal experiments were reviewed and approved by the review board for animal experiments of University Michigan (The Unit for Laboratory Animal Medicine, #A3114‐01, March 7, 2024). All animal experiments should comply with the ARRIVE guidelines and should be carried out in accordance with the U.K. Animals (Scientific Procedures) Act, 1986 and associated guidelines, EU Directive 2010/63/EU for animal experiments, or the National Institutes of Health guide for the care and use of laboratory animals (NIH Publications No. 8023, revised 1978).

## Supporting information

Supporting Information S1

Figure S1

Figure S2

Figure S3

## Data Availability

All data generated or analyzed during this study are included in this article and/or its supplementary material files. Further inquiries can be directed at the corresponding author.
