# Supplementary material for: Extracellular high mobility group box 1 as a UV‐induced alarmin driving cutaneous inflammation and photoaging via TLR4 signaling
Source: J Cell Commun Signal. 2026 Aug 27;20(3):e70109. doi: 10.1002/ccs3.70109 (PMC13522719; doi:10.1002/ccs3.70109)
Supplement: Supplementary file 1 [file CCS3-20-e70109-s002.docx]

Supplementary Figure 1: UV (2MED) irradiation induces HMGB1 release from human skin was confirmed using alternative HMGB1 antibody (Cell signaling Technology, Danvers, MA, USA). HMGB1 immunohistology from six individuals. . Data represent mean ± SEM. *p < 0.03, Scale bar = 100μm.

Supplementary Figure 2: (A) HMGB2 mRNA expression in keratinocytes (KCs) and fibroblasts (FBs). mRNA levels were quantified by qRT-PCR and normalized to 36B4 housekeeping gene. N=4. UV (2MED) irradiation induces HMGB2 release from human skin was confirmed by HMGB2 immunostaining from six individuals. Data represent mean ± SEM. *p < 0.03, Scale bar = 100μm.

Supplementary Figure 3: TAK-242 treatment does not alter TLR or RAGE gene expression. (A–D) Fibroblasts were pretreated with TAK-242 (100μM) for 1h before UV irradiation (50 mJ/cm²). Cells were harvested 24 h after UV irradiation, and TLR2 (A), TLR3 (B), TLR4 (C), and RAGE (D) mRNA levels were quantified by qRT-PCR and normalized to the 36B4 housekeeping gene. Data are presented as mean ± SEM (n= 4). (E–H) Mice were pretreated with vehicle (DMSO) or TAK-242 (3 mg/kg) for 2 h before UV irradiation (150 mJ/cm²). Skin samples were collected 24h after UV irradiation, and TLR2 (E), TLR3 (F), TLR4 (G), and RAGE (H) mRNA levels were quantified by qRT-PCR and normalized to the 36B4 housekeeping gene. Data are presented as mean ± SEM (n = 5 per group).
